# Antiviral activity and chemical characterization of Egyptian *Ziziphus spina-christi* against human respiratory viruses

**DOI:** 10.1038/s41598-026-47325-9

**Published:** 2026-04-19

**Authors:** Amany Elkhrsawy, Omnia Kutkat, Yassmin Moatasim, Mohamed S. Refaey, Ahmed A. Al-Karmalawy, Eman Elabd, Amr Keshta, Kamal Shaltout, Rabeh El-Shesheny

**Affiliations:** 1 Centre of Scientific Excellence for Influenza Viruses, National Research Centre, Giza, 12622 Egypt; 2 Botany Department, Faculty of Science, Tanta University, Tanta, 31527 Egypt; 3 Department of Pharmacognosy, Faculty of Pharmacy, University of Sadat City, Sadat City, 32897 Menoufia Egypt; 4 Department of Pharmacognosy and Natural Products, Faculty of Pharmacy, Menoufia National University, km Cairo-Alexandria Agricultural Road, Shibin Elkom, Menoufia Egypt; 5 Department of Pharmaceutical Chemistry, Faculty of Pharmacy, Horus University-Egypt, New Damietta, 34518 Egypt; 6 Department of Pharmaceutical and Drug Industries Research, National Research Centre, Giza, 12622 Egypt; 7 Environmental Sciences Council, Academy of Scientific Research and Technology, Cairo, 11516 Egypt

**Keywords:** *Ziziphus spina-christi (L.)*, Antiviral activity, SARS-CoV-2, MERS-CoV and influenza virus (H1N1), Biotechnology, Computational biology and bioinformatics, Drug discovery, Microbiology

## Abstract

Since acute respiratory infections are the most prevalent illnesses in humans and can develop into respiratory tract infections, they pose a serious threat to public health. One of the most crucial approaches to treating the respiratory tract is development of medications derived from natural sources. *Ziziphus spina-christi* (L.). is a well-known plant that has been used for centuries in the Middle East to treat a variety of illnesses, such as anti-inflammatory, anti-fungal, anti-microbial, and anti-viral activities. This study examined antiviral properties of *Z. spina-christi* leaves and fruits against three respiratory viruses: influenza virus H1N1, MERS-CoV, and SARS-CoV-2. The crude methanol extracts of the leaves and fruits were fractionated *n*-hexane, dichloromethane, and ethyl acetate, along with the remaining aqueous residues of the two parts to give their corresponding fractions. Chemical profiling of the crude extracts and fractions of the leaves and fruits was investigated using LC-ESI-MS/MS analysis. By applying in-silico molecular docking techniques, the compounds identified in the most active extract or fraction were examined to determine which phytocompounds might have antiviral properties and to predict how they function. The results showed that flavonoids, phenolic acids, saponins, sterols, and triterpenes and their glucosides are the major detected compounds in both leaf and fruit fractions and extracts. The most common phenolic in all six samples is caffeic acid, whereas the most common flavonoids in the fruits’ ethyl acetate fraction were quercetin, luteolin, taxifolin, and is quercetin. The common saponin chemicals found in leaf and fruit extracts and their fractions are lotoside II, Ziziphus saponin I, and betulinic acid. The results also exhibited that crude extract of leaves had high antiviral activity against H1N1 with IC_50_ equal 1.240 µg/ml and against SARS-CoV-2 with IC_50_ equal 7.397 µg/ml, by performed mode of action the results showed that the extracts had virucidal effect against three viruses by applying in-silico molecular docking techniques, binding energy with HA of H1N1 equal − 7.48 kcal/mol and binding energy with spike of SARS-CoV-2 equal − 10.18 kcal/mol. On the other hand, residual extract had antiviral efficacy against MERS-CoV with an IC_50_ equal to 1.501 µg/mL and binding energy with the spike equal to -8.19 kcal/mol. Our findings suggest that *Z. spina-christi* is a natural medication with potent antiviral effects.

**Supplementary Information:**

The online version contains supplementary material available at 10.1038/s41598-026-47325-9.

## Introduction

Acute respiratory infections represent a significant public health concern as they are the most common diseases affecting humans and can lead to severe complications when they progress to lower respiratory tract infections, a leading cause of morbidity and mortality worldwide. Emerging respiratory infectious diseases pose a serious threat to human health due to their high person-to-person transmissibility which can result in widespread illness and fatalities.

Influenza viruses and coronaviruses are among the primary viral pathogens responsible for respiratory infections in humans. Despite the availability of Food and Drug Administration (FDA)-approved antiviral drugs for influenza treatment, the virus’s high mutation rate and rapid evolution have led to the emergence of drug-resistant strains, thereby reducing the effectiveness of some currently available therapies^1^. The most recent addition to this group is Severe Acute Respiratory Syndrome Coronavirus 2 (SARS-CoV-2), the causative agent of the COVID-19 pandemic. First detected in November 2019 in Wuhan, China, SARS-CoV-2 spread rapidly worldwide posing an unprecedented global health threat^2^. Between 2019 and 2024, more than 775 million cases and over 7 million deaths were reported by the World Health Organization (WHO)^3^. Currently, there are no broad-spectrum antivirals or universal vaccines effective against all coronaviruses. While specific treatments for SARS-CoV-2 infection are available, their use is restricted to certain adult populations, and some have been associated with adverse reactions^4^. Research activities continue to explore the genomic determinants of pathogenicity in SARS-CoV-2 and other human coronaviruses to better understand their infection mechanisms and identify potential therapeutic targets.

Although preventive vaccines are being developed, the continuous evolution of SARS-CoV-2 and influenza viruses poses an important challenge, particularly due to the emergence of drug-resistant mutants especially when using viral enzyme-specific inhibitors whose effectiveness might decline over time^5^, and the adverse health effects associated with some approved antiviral drugs. As a result, the search for new, safe, and effective antiviral treatments remains a critical priority. Given the global burden of respiratory infectious diseases, particularly those caused by influenza and coronaviruses, there is an urgent need to develop affordable and widely accessible treatments suitable for different age groups and economic settings.

One promising avenue for addressing this challenge is plant-based medicine. Nature has long served as a source of therapeutic substances, and many modern pharmaceuticals have been derived from natural sources. In addition, medicinal plants have played a significant role in the treatment of cancer, inflammatory diseases, and infectious diseases over the past four decades. Their therapeutic potential lies in their rich diversity of secondary metabolites and bioactive compounds, many of which exhibit antiviral, anti-inflammatory, and immune-modulating properties. Moreover, medicinal plant-based treatments are often associated with low toxicity, commercial availability, and minimal side effects, making them a promising alternative to synthetic drugs^6^.

Egypt’s habitat diversity, shaped by its unique geographical, physiographic, edaphic, and climatic conditions, is reflected in its rich plant biodiversity. The most common wild medicinal plants found in Egypt are *Acacia nilotica*,* Achillea fragrantissima*,* Adiantum capillus- veneris*,* Origanum syriacum*,* and Moringa pereg.* One of the most promising and available medicinal plants in Egypt is *Ziziphus spina-christi*, commonly known as the Nabq or Sidr tree. This plant belongs to Rhamnaceae family and it is spiny shrub, the tree can reach 5–10 m in height with a trunk diameter of up to 45 cm. Its leaves are simple, smooth, narrow, pointed at the tip, crenate and with an obtuse apex. The branches are glabrous with stipular spines and the shorter branches are curved. It’s cultivated in dry and warm regions and distributed in West Asia and North Africa (occupies all phytogeographical areas in Egypt). This tree contains a variety of bioactive compounds such as flavonoids, terpenoids, saponins, alkaloids, phenolics, amino acids, and essential oils. *Ziziphus* extracts were used in ancient Egyptian medicine as remedies for swelling, pain, and fever, suggesting anti-inflammatory properties^7^. The leaves of this plant have been found to contain anti-inflammatory, anti-fungal, and anti-microbial agents^8–12^. Additionally, *Ziziphus spina-christi* extracts have demonstrated anti-cancer^13^ activity and antiviral activities against respiratory viruses such as influenza, with low cytotoxicity in various cell types^14^.

The identification of antiviral mechanisms of these natural agents has shed light on how these agents interact with the viral life cycle, including viral entry, replication, assembly, and release, as well as their ability to target virus–host interactions. Several plant-based extracts have demonstrated notable antiviral properties against respiratory viruses. For instance, the liquid extract of *Rubini elderberry* and *Pelargonium sidoides roots* have been shown to display an inhibitory effect on the propagation of human pathogenic influenza viruses^15,16^. Similarly, the aqueous extract of *Houttuynia cordata* has been found to exhibit several antiviral mechanisms against SARS-CoV, including inhibition of the viral 3CL protease and blocking the viral RNA-dependent RNA polymerase activity^17^.

Despite the large body of evidence describing the medicinal and pharmaceutical properties of *Ziziphus spina-christi* extracts, and their role as indirect antivirals through boosting cellular activity and anti-inflammatory activity, the exact direct role of different leaf and fruit extracts and their constituent bioactive compounds in inhibiting respiratory virus replication within the viral life cycle remains unclear. Here, we aimed to investigate the antiviral potential of different extracts of *Ziziphus spina-christi* against respiratory viruses as a broad antiviral activity through in vitro testing in cell culture, followed by predicting the most promising bioactive ingredient in the most potent extracts through in-silico prediction. Network medicine and artificial intelligence (AI) technologies offers a powerful approach to accelerate the discovery of new drug alternatives^18^. Computational studies such as molecular docking are one of the crucial tools in the process of drug discovery as they help save effort, time, and cost while enabling the prediction of binding affinity between candidate compounds and molecular targets^19,20^.

## Materials and methods

### Plant materials

Fresh aerial parts (leaves and fruits) of *Ziziphus spina-christi* were collected in fall 2022 from Gharbia Governorate, Egypt, following permission from the local owner. The plant was authenticated by Prof. Dr. Mona M. Marzouk, professor of chemistry of natural products and plant chemosystematics. A voucher specimen (No. M292) was deposited at the herbarium of National Research Centre (CAIRC), Giza, Egypt. The plant materials were shade-dried and finely powdered for the extraction process.

### Extraction and fractionation

The powdered, dried leaves and fruits (~ 5 g) of *Z. spina-christi* were extracted with 70% aqueous methanol by maceration at room temperature till exhaustion (50 mL × 3). The combined alcoholic extracts of each part of the plant were concentrated under reduced pressure at 40 ℃ and left to dry yielding 10 g each of leaf and fruits extracts. The dried leaf and fruit extracts were suspended in distilled water (100 mL) and successively partitioned between *n*-hexane, dichloromethane, and ethyl acetate (50 mL × 3). Each phase was concentrated under reduced pressure separately to give their corresponding fractions together with the remaining aqueous residues.

### Mass-mass spectrometry analysis

Sample analysis was performed using Liquid Chromatography–Electrospray Ionization–Tandem Mass Spectrometry (LC-ESI-MS/MS) with an ExionLC AC system for separation and SCIEX Triple Quad 5500 + MS/MS + MS/MS system equipped with an electrospray ionization (ESI) for detection. The separation was performed using an Ascentis^®^ Express 90 Å C18 Column (2.1 × 1_50_ mm, 2.7 μm). The mobile phase consisted of two eluents A: 5 mM ammonium format (pH 8) and B: acetonitrile (LC grade), and the gradient elution conditions were: 5% B at 0–1 min, 5-100% B from 1 to 20 min, 100% B from 20 to 25 min, 5% at 25.01, and 5% from 25.01 to 30 min. The flow rate was 0.3 ml/min and the injection volume was 5 µl. For MS/MS analysis, negative ionization mode was applied with a scan (EMS-IDA-EPI) from 100 to 1000 Da for MS1 with the following parameters: curtain gas: 25 psi; IonSpray voltage: -4_50_0; source temperature: _50_0°Ci; ion source gases 1 and 2 were 45 psi each and from 50 to 1000 Da for MS2 with a delustering potential of -80 and a collision energy of -35. Compound identification was performed using MS-DIAL using Respect library.

### Cells and viruses

African green monkey kidney (Vero E6) (ATCC No. CRL-1586) and Madin-Darby canine kidney (MDCK) (ATCC CCL-34) cell lines were kindly provided by Dr. Richard Webby, Department of Host-Microbe Interactions, Division of Virology, St. Jude Children’s Research Hospital, USA. The cells were grown in Dulbecco’s modified Eagle’s medium (DMEM Gibco, Thermo Fisher Scientific, Waltham, MA, USA) containing 10% fetal bovine serum (FBS Gibco, Thermo Fisher Scientific, Waltham, MA, USA) supplemented with 2% from antibiotic antimycotic (Gibco, Thermo Fisher Scientific, Waltham, MA, USA). For virus propagation in MDCK cells, DMEM (without FBS) supplemented with 2 µg/mL tosyl phenylalanyl chloromethyl ketone (TPCK)–trypsin (Sigma) and bovine serum albumin (BSA) was used as the virus culture medium.

VERO E6 cell lines were used to propagate and titrate SARS-CoV-2 [CoV-19/Egypt/NRC-03/2020 (GISAID number: EPI_ISL_430819)]^21^, and MERS-CoV [NRCE-HKU270 (Genbank Accession: KJ477103.2)]^22^, while MDCK cell lines were used to propagate and titrate H1N1 [A/California/04/2009 (NCBI: txid641_50_1)]. The propagation and titration of SARS-CoV-2 and MERS-CoV viruses were performed in class III biosafety cabinet. Virus titers were measured using the 50% tissue culture infectious doses (TCID_50_) assay^23,24^. Cells were seeded in 96-well plates at a density of 1.0 × 10^4^ cells/well and incubated at 37 °C overnight. Virus stocks were serially diluted ten folds, then cells were inoculated in quaternity with 200 µl diluted virus sample. After 3 days of incubation at 37 °C, the TCID_50_ was determined using the Reed and Muench method, using HA assay for influenza H1N1 virus, and microscopical examination for SARS-CoV-2 and MERS-CoV.

### Cytotoxicity assay on cells by crystal violet

Cytotoxicity of the different leaf and fruit extracts was determined by calculating the 50% cytotoxic concentration (CC_50_) as previously described^25^. In 96-well tissue culture plates, 2.4 × 10^4^ of Vero-E6 or MDCK cells were seeded per well and incubated overnight. Two-folds serial dilutions of extracts were used to treat cells and incubated for 72 h. Cells were then fixed with 4% paraformaldehyde and stained with 0.1% crystal violet. Absolute methanol was used to dissolve the dye for optical density measurement at λ = 570 nm. The nonlinear regression analysis was used to calculate CC_50_ values using GraphPad Prism software version 8.01 (San Diego, CA, USA).

### Antiviral activity

The 50% inhibitory concentration (IC_50_) was performed to determine the concentrations that reduce the virus-induced cytopathic effect (CPE) by 50%, as previously described^26–28^. Remdesivir and Oseltamivir were kindly provided by Egyptian Drug Authority (EDA). Remdesivir wasused as control drugs against SARS-CoV-2, MERS-CoV, and oseltamivir was used against H1N1 influenza virus. Preparation and final concentrations of control drugs were done according to previous studies^29–32^. In 96-well tissue culture plates, two-folds serial dilutions of each extract was treated with 100 TCID_50_/well of the corresponding virus and incubated for 1 h at room temperature (RT), then monolayers of Vero-E6 and MDCK cells were treated with the virus-extract mixture and incubated at 37 °C in a 5% CO_2_ incubator for 72 h. Cells were fixed with paraformaldehyde for 2 h and stained with 0.1% crystal violet for 15 min at RT. The crystal violet dye was then dissolved using 100 µl of absolute methanol per well and the optical density of the color is measured at 570 nm using plate reader. The IC_50_ of the compound was defined as the concentration of the compound required to reduce the virus-induced cytopathic effect (CPE) by 50% relative to the virus control and it is important for calculating Safety Index (SI) according to the following equation: SI=CC_50_/IC_50_.

### Mode of action for viral inhibition

The mode of action of the selected potent extracts against the three viruses was tested at three different stages of the virus propagation cycle and based on three main possible modes of action which were performed under the same conditions from passage of cells and titration of viruses: (1) Inhibition of budding and viral replication, (2) The ability of extracts to inhibit the attachment of the virus to cells-membrane fusion known as blocking the viral entry (viral adsorption), and (3) The direct effect of the extracts to inactivate the virus viability (virucidal activity). Additionally, the above-mentioned mode of action could account for the recorded antiviral activities either independently, or in combinations.

#### The viral replication assay^33^

This step was performed by viral incubation with the cells for 1 h. After incubation, the unabsorbed virus was removed, and the cell monolayers were washed twice to eliminate residual viral particles before treating the cells with different concentrations of the plant extract.

#### Viral adsorption assay^34^

In this step, extracts were added firstly on the host cells for 1 h before starting the viral-cell incubation, the cell monolayers in six-well plate were treated with various concentrations of the plant extract (100 µL per well) and were incubated at 37 °C in a 5% CO_2_ atmosphere for 2 h. After incubation, the remaining unabsorbed extract was removed, and treated cells were infected with corresponding virus before addition of the agarose overlay.

#### Virucidal assay^35^

This step aimed to evaluate the direct effect of the plant extract on viral infectivity. Different concentrations of the selected plant extracts were treated with the virus and incubated at 37 °C for 1 h before adding the mix to cells monolayers. Meanwhile, a six-well plate containing confluent cells was prepared two days prior. The growth medium was removed, and 200 µL of each virus-extract mixture was added to the cell monolayers.

For the three different mechanisms, the plates were incubated at 37 °C until viral plaques formed after the addition of agarose overlay. The plaques were fixed for 2 h using 10% formalin solution and then stained with 0.1% crystal violet for visualization. To ensure accurate assessment of the antiviral effects, the experiments included untreated cell controls, virus-only controls. The data were normalized against the control values; a control group of uninfected cells was included in each experiment. This control group allowed direct comparison, ensuring that the observed plaques were specifically due to viral infection rather than spontaneous cell death, environmental factors, or morphological changes unrelated to viral replication. The result represented on GraphPad Prism version 8.0.2 was used to calculate statistical significance through 2-way ANOVA test followed by Tukey multiple comparison tests, with a 95% confidence interval.

### Docking studies

AutoDock Vina and PyMol software^36,37^ have been applied to predict the antiviral activity of the *Ziziphus spina Christi*. Accordingly, the extracts that showed better activities against the H1N1, MERS-CoV-2, and SARS-CoV-2 were further investigated through molecular docking. The identified compounds were transferred from ChemDraw to the working area of the drug design program to be optimized for partial charges and energy minimization^38^. Moreover, the target protein receptors, HA of H1N1 (PDB ID: 6WCR), NA of H1N1 (PDB ID: 3CKZ), the spike of MERS-CoV-2 (PDB ID: 7 × 27), and the spike of SARS-CoV-2 (PDB ID: 6VW1) were downloaded from the Protein Data Bank. Each receptor was corrected, energy minimized, and 3D hydrogenated^39^. Four different molecular docking processes were performed to compare the binding affinities of the identified compounds towards the three target receptors. Additionally, two validation processes were carried out by redocking the co-crystallized inhibitors of the HA and NA of H1N1, and the obtained RMSD values (< 2 Å) showed the validity of the applied software^40^(Supplementary Data).

## Results

### Metabolic profiling of *Z. spina-christi* leaf and fruit extracts using LC-ESI-MS/MS

After the extraction of leaves and fruits, and based on the exact mass, the observed spectra fragmentation patterns, and literature data, the structural characterizations of chemical composition in the crude methanol extracts, ethyl acetate fractions, and remaining aqueous residues for both leaves and fruits of *Z. spina christi* were conducted. Using MS/MS fragmentation pattern in negative mode, numerous compounds from different classes of secondary metabolites were identified. The detected compounds’ structures for each extract/or fraction are presented in the supplementary Material (Fig S1—S6). Molecular ion, retention time, and MS/MS data of identified compounds are provided in supplementary Tables S1—S6.

### Cytotoxicity and antiviral activity of *Ziziphus spina-christi* extracts against SARS-CoV-2 and MERS-CoV

**Fig. 1:**
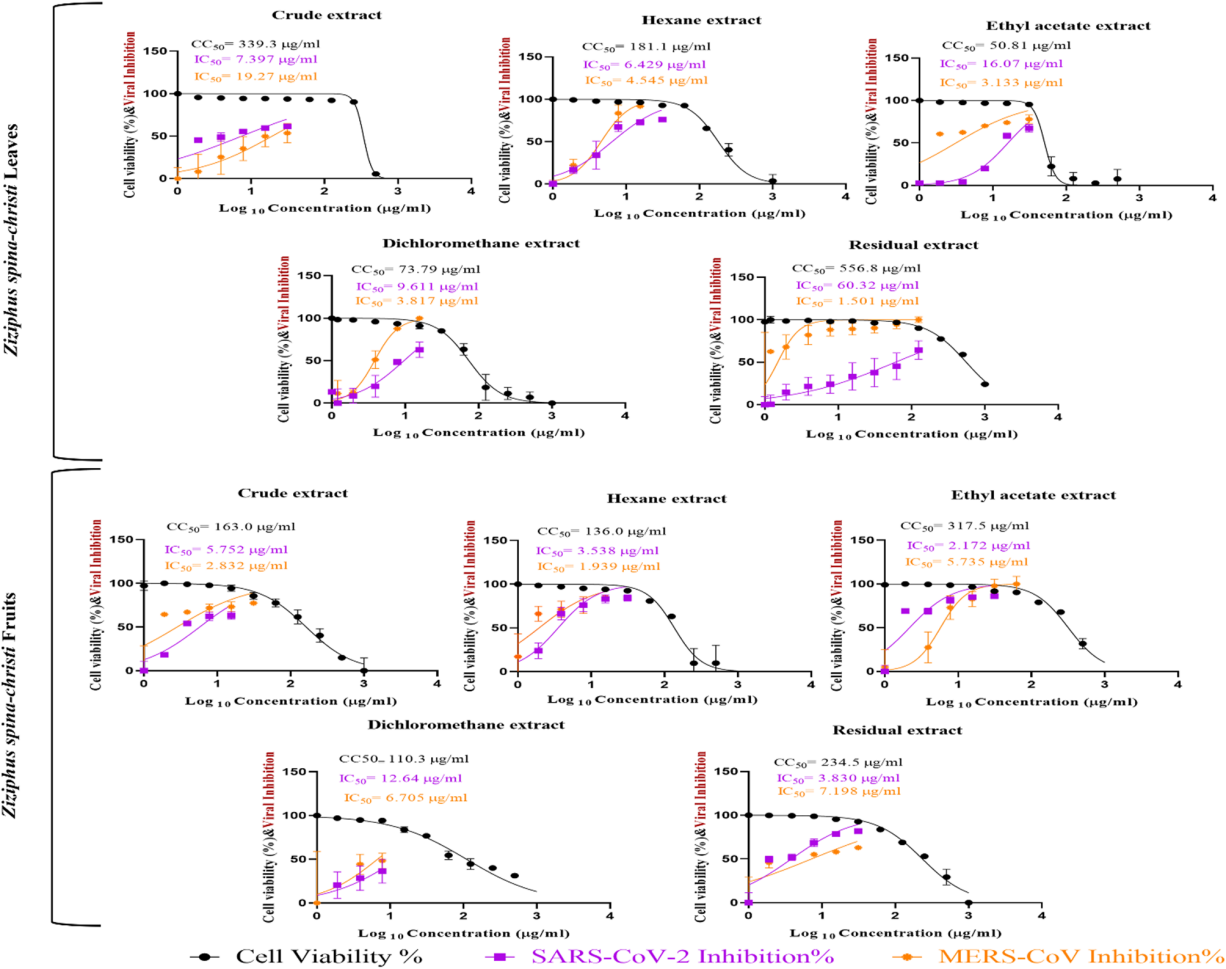
Cytotoxicity Concentration 50% (CC_50_) of leaf and fruit extracts of *Ziziphus spina-christi* on Vero E6 and Inhibitory Concentrations 50% (IC_50_) against SARS-CoV-2 and MERS-CoV, Plotting log inhibitor versus normalized response (varying slope) allowed for the nonlinear regression analysis using GraphPad Prism software (version 8.01) to get the values of CC_50_ and IC_50_.

**Fig. 2:**
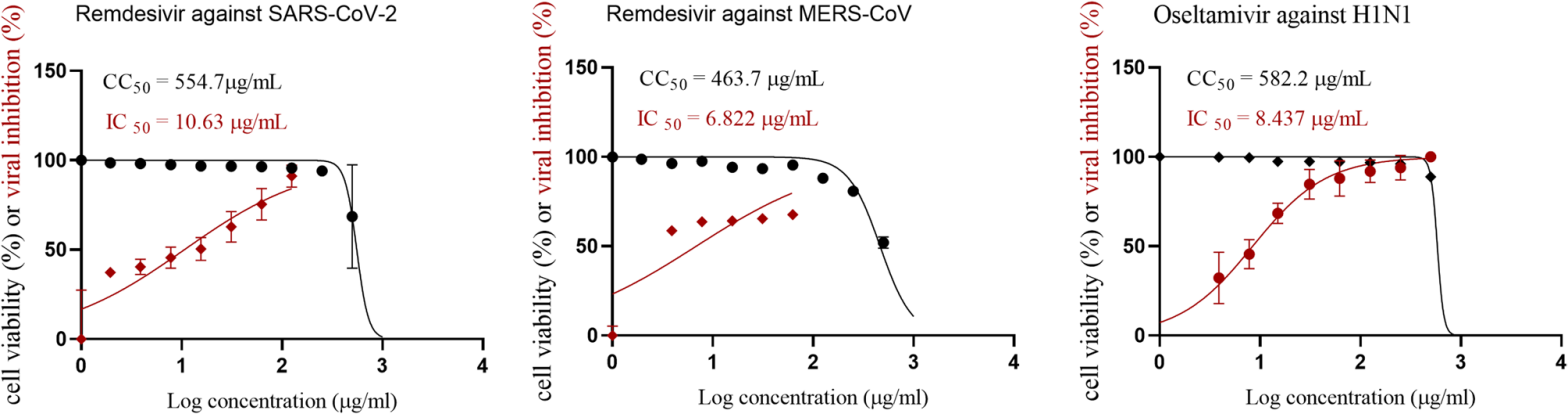
Cytotoxicity Concentration 50% (CC_50_) and Inhibitory Concentrations 50% (IC_50_) of control remdesivir against SARS-CoV-2 and MERS-CoV on Vero E6, and Oseltamivir against H1N1 influenza viruses, Plotting log inhibitor versus normalized response (varying slope) allowed for the nonlinear regression analysis using GraphPad Prism software (version 8.01) to get the values of CC_50_ and IC_50_.

The safe dose and antiviral activity of the five extracts from *Ziziphus spina-christi* from both fruits and leaves on the Vero E6 cell line were assessed and illustrated in Fig. 1; Tables 1 and 2. The CC_50_ of each fruit extract were determined to be 136.0 µg/ml for the hexane extract, 317.5 µg/ml for the ethyl acetate extract, 163.0 µg/ml for the crude fruit extract, 110.3 µg/ml for the dichloromethane extract, and 234.5 µg/ml for the residual fruit. For leaves, the CC_50_ for each extract were as follows: 181.1 µg/ml for the hexane extract, 50.81 µg/ml for the ethyl acetate extract, 339.3 µg/ml for the original leaf extract, 73.79 µg/ml for the dichloromethane extract, and 556.8 µg/ml for the residual leaves. Remdesivir was used as control drug against SARS-CoV-2 and MERS-CoV (Fig. 2).

The antiviral activity against SARS-CoV-2 and MERS-CoV revealed that all extracts exhibited positive antiviral activity. Among all leave extracts, the crude leaf extract showed the highest anti-SARS-CoV-2 activity with an IC_50_ value of 7.4 µg/ml and SI of 45.9, while the residual leaf extract showed the highest anti-MERS-CoV activity with an IC_50_ value of 1.5 µg/ml and SI of 371.0. Among the fruit extracts, the ethyl acetate extract showed the highest anti-SARS-CoV-2 activity with an IC_50_ value of 2.2 µg/ml and SI of 146.2, while the hexane extract showed the highest anti-MERS-CoV activity with an IC_50_ value of 1.9 µg/ml and SI of 70.1.

**Table 1:** (CC_50_), (IC_50_) and (SI) of leaf and fruit extracts of *Ziziphus spina-christi* against SARS-CoV-2, using Vero E6 cell line.

Plant part	Extract type	Cyto-toxicity concentration (CC_50_) µg/ml	SARS-CoV-2 inhibition concentration (IC_50_) µg/ml	Selectivity index (CC_50_/IC_50_)
*Ziziphus spina-christi* Leaves	Crude	339.3	7.4	45.9
	Hexane	181.1	6.4	28.2
	Ethyle acetate	50.8	16.1	3.2
	Dichloromethane	73.8	9.6	7.7
	Residual	556.8	60.3	9.2
*Ziziphus spina-christi* Fruits	Crude	163.0	5.7	28.3
	Hexane	136.0	3.5	38.4
	Ethyle acetate	317.5	2.2	146.2
	Dichloromethane	110.3	12.6	8.7
	Residual	234.5	3.8	61.2

**Table 2:** (CC_50_), (IC_50_) and (SI) of leaf and fruit extracts of *Ziziphus spina christi* against MERS-CoV, using Vero E6 cell lines.

Plant part	Extract type	Cyto-toxicity concentration (CC_50_) µg/ml	*MERS-CoV* inhibition concentration (IC_50_) µg/ml	Selectivity index (CC_50_/IC_50_)
*Ziziphus spina-christi* Leaves	Crude	339.3	19.3	17.6
	Hexane	181.1	4.5	39.8
	Ethyle acetate	50.8	3.1	16.2
	Dichloromethane	73.8	3.8	19.3
	Residual	556.8	1.5	371.0
*Ziziphus spina-christi* Fruits	Crude	163.0	2.8	57.5
	Hexane	136.0	1.9	70.1
	Ethyle acetate	317.5	5.7	55.4
	Dichloromethane	110.3	6.7	16.5
	Residual	234.5	7.2	32.6

### Cytotoxicity and antiviral activity of *Ziziphus spina-christi* extracts against human influenza H1N1 virus

**Fig. 3:**
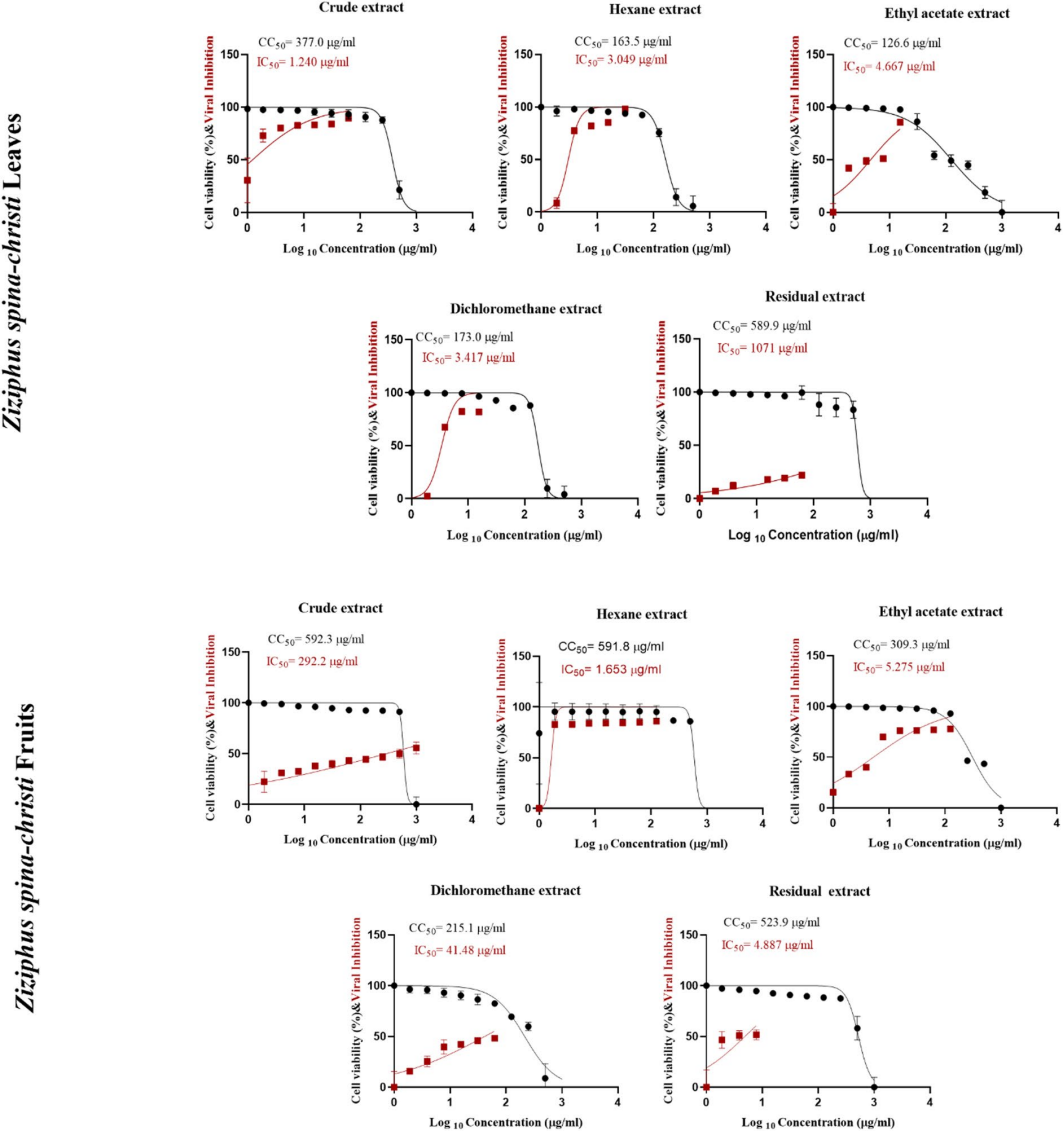
Cytotoxicity Concentration 50% (CC_50_) of leaf and fruit extracts of *Ziziphus spina-christi* on MDCK cell line and Inhibitory Concentrations 50% (IC_50_) graph against H1N1 virus, Plotting log inhibitor versus normalized response (varying slope) allowed for the nonlinear regression analysis using GraphPad Prism software (version 8.01) to get the values of CC_50_ and IC_50_.

Oseltamivir was used as control drug against H1N1 influenza virus (Fig. 2). The cytotoxic effects of the five extracts from *Ziziphus spina-christi* fruits on the MDCK cell line were assessed and displayed in Fig. 3; Table 3. The CC_50_ for each fruit extract were as follows: 591.8 µg/ml for the hexane extract, 309.3 µg/ml for the ethyl acetate extract, 592.3 µg/ml for the crude fruit extract, 215.1 µg/ml for the dichloromethane fruit extract, and 523.9 µg/ml for the residual fruit. The antiviral activity of fruit extracts against the human influenza virus H1N1 indicated that the most potent was hexane extract, with IC_50_ values of 1.7 µg/ml and SI of 358.0. However, the crude fruit extract showed very low antiviral activity against H1N1.

The CC_50_ of each leaf extract was determined as follows: 163.5 µg/ml for the hexane extract, 126.6 µg/ml for the ethyl acetate extract, 377.0 µg/ml for the original leaf extract, 173 µg/ml for the dichloromethane extract, and 589.9 µg/ml for the residual leaves. The antiviral activity of leaf extracts against human influenza virus H1N1 revealed that the highest ant-H1N1 activity was exhibited by the crude extract, with IC_50_ values of 1.2 µg/ml and SI of 304.0. However, the residual leaves exhibited no antiviral activity against H1N1.

**Table 3:** (CC_50_), (IC_50_) and (SI) of leaf and fruit extracts of *Ziziphus spina-christi* against H1N1 Human influenza virus, using MDCK cell lines.

Plant Part	Extract type	Cyto-toxicity concentration (CC_50_) µg/ml	*H1N1* inhibition concentration (IC_50_) µg/ml	Selectivity index (CC_50_/IC_50_)
*Ziziphus spina-christi* Leaves	Crude	377.0	1.2	304.0
	Hexane	163.5	3.1	53.6
	Ethyle acetate	126.6	4.7	27.1
	Dichloromethane	173.0	3.4	50.6
	Residual	589.9	1071.0	0.6
*Ziziphus spina-christi* Fruits	Crude	592.3	292.2	2.0
	Hexane	591.8	1.7	358.0
	Ethyle acetate	309.3	5.3	58.6
	Dichloromethane	215.1	41.5	5.2
	Residual	523.90	4.9	107.2

### Mode of antiviral activity of the most active extracts

**Fig. 4:**
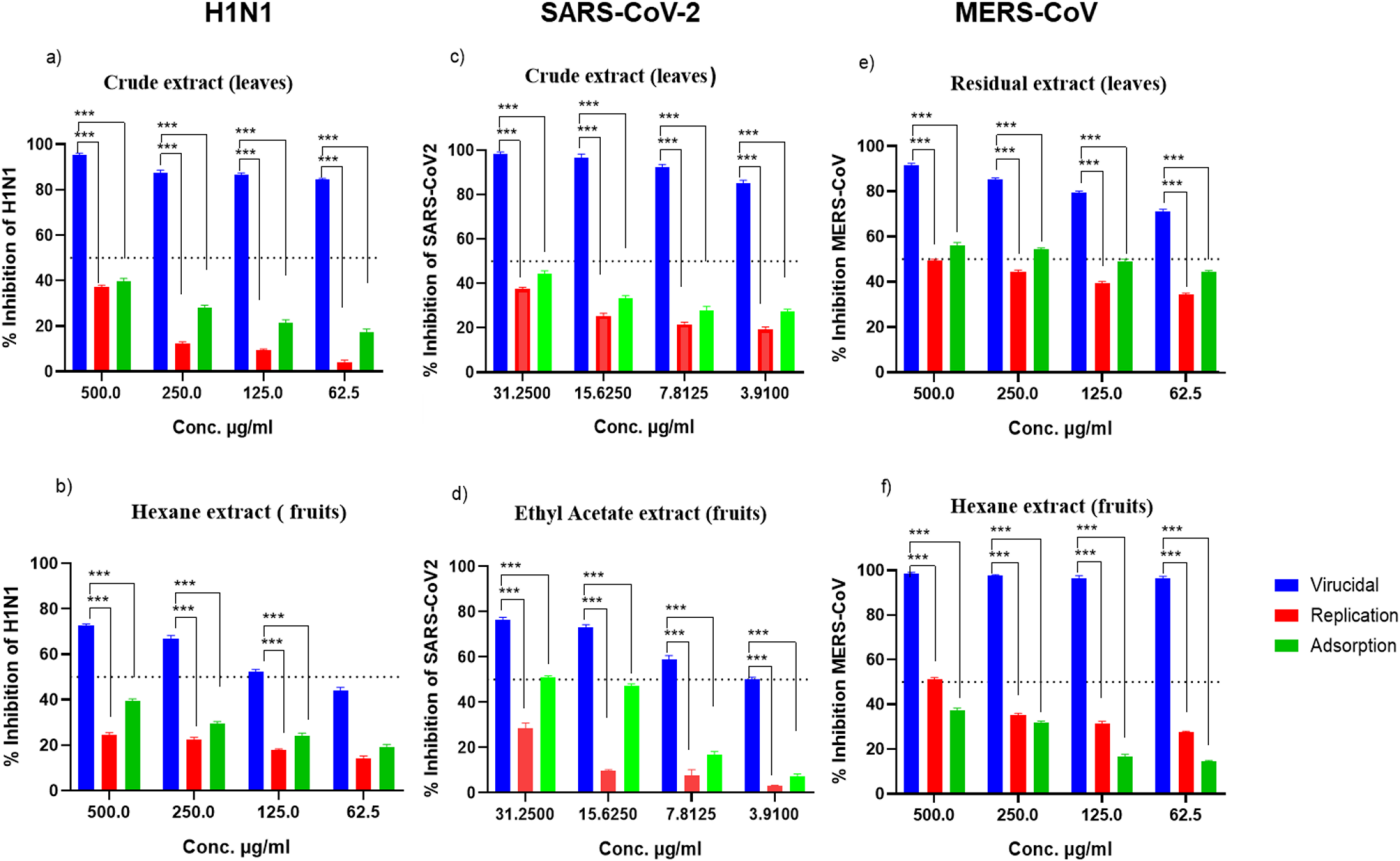
Mode of action of two extracts of *Ziziphus spina-christi* against H1N1 (**a**) residual of leaves, (**b**) hexane fruit extract; SARS-CoV-2 (**c**) original leaf extracts, (**d**) ethyl acetate fruit extract; and MERS-CoV, (**e**) residual of leaves, and (**f**) hexane fruit extract. Statistical significance is calculated through 2-way ANOVA tests followed by Tukey multiple comparisons test, 95% confidence interval, *** refers to *p* ≤ 0.001.

To investigate the stage of the viral replication cycle where the lowest IC_50_ (and highest SI) extracts exhibited antiviral activity, the extracts that showed highest antiviral activity against the three tested viruses underwent viral inhibition assay using plaque reduction assay in dose-dependent manner. Interestingly, all tested extracts showed significantly higher remarkable antiviral activity and inhibition in the virucidal step, by directly affecting the viral surface protein binding to the cellular proteins and viral adsorption as shown in Fig. 4.

Original leaf extracts and hexane fruit extracts of *Ziziphus spina-christi* were the most potent extracts with anti-human influenza virus H1N1 activity. The virucidal inhibition was around 95% with original extract and around 75% with hexane fruit extract. On the other hand, the inhibition by adsorption and replication was not more than 40% for the two extracts.

Original leaf extracts and ethyl acetate fruit extracts were the most potent extracts that had anti-SARS-CoV-2 virus activity; the results showed that, the virucidal inhibition was more than 95% with original extract and around 75% with ethyl acetate fruit extract. On the other hand, the inhibition didn’t exceed 50% by adsorption and replication mechanisms. Residual leaves and hexane of fruit extracts were the most potent extracts against MERS-CoV; the virucidal inhibition was around 90% more than effect on adsorption and replication where inhibition ranged from 40 to 60%.

### Docking studies

The identified compounds from the extracts with the lowest IC_50_ and highest SI were tested through in silico analysis to identify the most potent compound in each extract. The identified compounds from both the original leaf extract and the residual of leaves showing better activities against the H1N1 and MERS-CoV-2, respectively, were further investigated through molecular docking. The target protein receptors, HA of H1N1 (PDB ID: 6WCR), NA of H1N1 (PDB ID: 3CKZ), the spike of MERS-CoV-2 (PDB ID: 7 × 27), and the spike of SARS-CoV-2 (PDB ID: 6VW1) were selected for four different molecular docking processes. Interestingly, both lotoside II and hydroxygenistein methyl ether malonylhexoside were found to be the superior candidates against the four targets and accordingly were selected for further investigation (Table 4).

Concerning the HA of H1N1 docking (PDB ID: 6WCR); lotoside II (Binding score = -7.48 kcal/mol and RMSD = 1.74 Å) formed three hydrogen bonds with His38, Asn53, and Ser291. However, hydroxygenistein methyl ether malonylhexoside (Binding score = -6.79 kcal/mol and RMSD = 1.23 Å) formed one hydrogen bond and one hydrogen-pi bond with His38. The co-crystallized inhibitor of the HA receptor of H1N1 showed a binding score of -6.23 kcal/mol and an RMSD of 1.20 Å. On the other side, For the NA of H1N1 (PDB ID: 3CKZ) results, lotoside II (Binding score = -8.00 kcal/mol and RMSD = 1.90 Å) represented five hydrogen bonds with Asp151 (2), Trp178, Glu276, Glu277, and Arg371. However, hydroxygenistein methyl ether malonylhexoside (Binding score = -7.72 kcal/mol and RMSD = 1.63 Å) formed two hydrogen bonds with Glu277 and Arg371. The co-crystallized inhibitor of the NA receptor of H1N1 showed a binding score of -9.79 kcal/mol and an RMSD of 1.01 Å.

Furthermore, analyzing the docking towards the spike protein of MERS-CoV-2 (PDB ID: 7 × 27); lotoside II (Binding score = -8.19 kcal/mol and RMSD = 1.84 Å) formed one hydrogen bond with Asp902. Besides, hydroxygenistein methyl ether malonylhexoside (Binding score = -8.27 kcal/mol and RMSD = 1.18 Å) bound with Asp902 and Glu944 with two hydrogen bonds. However, the docking results of the SARS-CoV-2 spike (PDB ID: 6VW1) showed that lotoside II (Binding score = -10.18 kcal/mol and RMSD = 1.86 Å) formed three hydrogen bonds with Glu145, Glu402, and Arg518. While hydroxygenistein methyl ether malonylhexoside (Binding score = -8.84 kcal/mol and RMSD = 1.90 Å) bound with Glu145, His345, and Ala348 with three hydrogen bonds. Also, it formed two pi-pi interactions with His345 and Phe504.

Based on the previous results, we can conclude that the original leaf extract and the residual leaf extract of *Ziziphus spina-christi* (especially lotoside II and hydroxygenistein methyl ether malonylhexoside members) may represent very promising inhibitors against H1N1, MERS-CoV-2, and SARS-CoV-2.

**Table 4:**
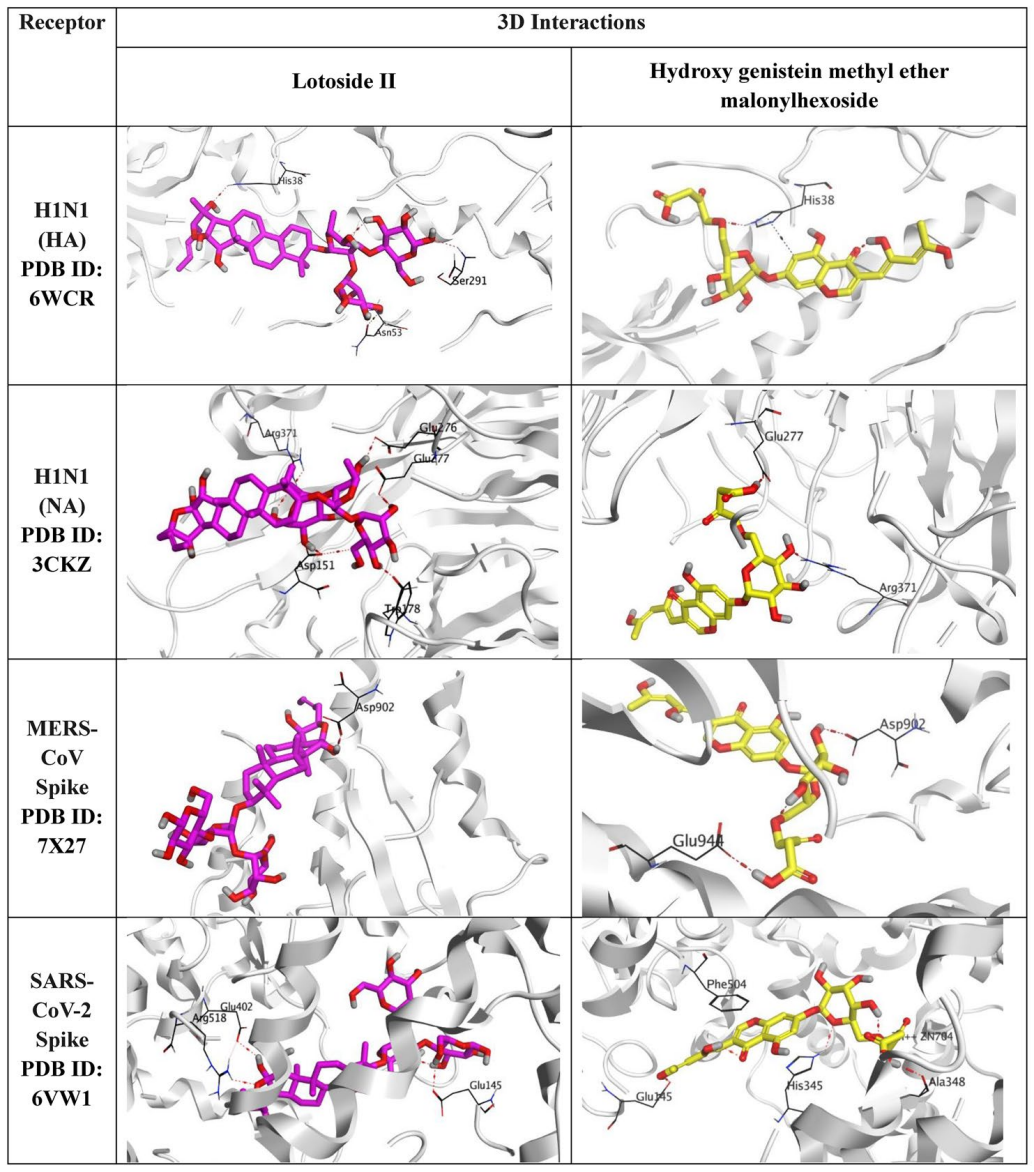
3D binding interactions of lotoside II and hydroxygenistein methyl ether malonylhexoside identified compounds to the HA and NA target receptors of H1N1, MERS-CoV-2 spike, and SARS-CoV-2.

## Discussion

*Ziziphus spina-christi* is a tree that produces edible fruits known as Nabq. It belongs to Rhamnaceae family that is adapted to grow in warm or hot climates. It is one of the oldest medicinal plants used in ancient Egypt medicine for its anti-inflammatory properties^7^. Its known to be rich in chemical compounds, and nowadays, leaf, fruit, and seed extracts have been reported to possess several pharmacological benefits, including management of diabetes and treatment of malaria, digestive issues, typhoid, liver problems, weakness, skin infections, urinary disorders, obesity, diarrhea, and antimicrobial activity (antifungal, antiviral, and anti-bacterial activity)^9^. In this study, we tested the antiviral potential of different fruit and leaf extracts of *Ziziphus spina-christi* and used the docking study to predict the main potent compounds that may inhibit the replication of three viruses, Human influenza H1N1, MERS-CoV-2 spike, and SARS-CoV-2. Five types of extracts from leaves and fruits were tested, (crude original, Hexane, Ethyle acetate, Dichloromethane, and the residual extracts). Each extract was subjected to mass spectrophotometry to identify its chemical compounds.

Flavonoids, phenolic acids, saponins, sterols, and triterpenes and their glucosides were the major detected compounds in both leaf and fruit fractions and extracts. Of these phenolics, caffeic acid (*m/z* 179.00 [M-H]^−^) was the most predominant in all six samples in negative ion mode, while its glucoside, caffeic acid 4-*O*-glucoside (*m/z* 341.01 [M-H]^−^) was identified in methanol extract of both leaves and fruits beside the remaining aqueous fraction of the fruits. These findings are in agreement with previous studies on leaf extract of Z. *spina-christi*^41^ and *Z. lotus* fruits^42^. Additionally, caffeoylquinic acid (*m/z* 353 [M-H]^−^) was detected in all tested plant sample extracts and fractions except the ethyl acetate fraction of the fruits. This compound and its analogues have been previously isolated from *Z. spina-christi* leaf extracts^43^. Among the flavonoid, quercetin, luteolin, taxifolin, isoquercetin, quercetin 3-*O*-(2-*O*-rhamnosyl-arabinoside), 5,7-Dihydroxy- 8,3’,5’ trimethoxy-flavone, and quercetin-3-*O*-α-L-arabinopyranosyl-(1→2)-α-L-rhamnopyranoside were predominantly detected in the ethyl acetate fraction of the fruits. This is in accordance with the previous studies^44–46^ where quercetin-3-*O*-α-L-arabinopyranosyl-(1→2)-α-L-rhamnopyranoside was isolated from *Z. spin-cristi* leaves, and isoquercitrin and quercetin were detected in the methanolic fruit extract. Moreover, the isoflavonoid compound hydroxygenistein methyl ether malonylhexoside (*m/z* 547 [M-H]^−^) was identified in all leaf and fruit samples except the remaining aqueous fraction of the fruits. The dammarane saponin, lotoside II (*m/z* 957 [M-H]^−^), the triterpenoid saponin *Z*izyphus saponin I (*m/z* 911 [M-H]^−^), and the triterpenoid compound betulinic acid (*m/z* 455 [M-H]^−^) were the common saponin compounds present in both leaf and fruit extracts and their fractions. Previous studies reported the isolation of lotoside II from root bark of *Z. lotus*, while ziziphus saponin I was isolated from the methanol extract of *Z. mauritiana*^47^. Additionally, chloroform extracts of *Z. jujuba* fruits were rich in betulinic acid and other triterpenic acids^48^. Ceanothic acid (*m/z* 485 [M-H]^−^), a ring-A homologue of betulinic acid, was identified in the methanol extract and ethyl acetate fraction of both leaves and fruits, but it wasn’t detected in the aqueous fractions. This compound has previously been reported in fruits of *Z. jujuba* and *Z. jujuba* var. *spinosa*^49^, as well as in methanolic leaf extracts of *Z. mauritiana* Lam., *Z. spina-christi* (L.), and *Z. jujuba*^50^. The sterol compound, stephanol, (*m/z* 397 [M-H]^−^) was identified in the fruit methanol extract and its fractions, but not in the methanol leaf extracts or fractions.

The extracts were then titrated in a dose-dependent manner in VERO and MDCK cell lines, to determine the safe concentration that could be used safely in subsequent assays. In the anti-coronavirus assays, the crude leaf extract (SI = 45.9) and the ethyl acetate fruit extract (SI = 146.3) were the most potent in vitro against SARS-CoV-2, while the residual leaf extract (SI = 371.0) and the hexane fruit extract (SI = 70.1) were the most potent in vitro against MERS-CoV. On the hand, the antiviral activity against the human influenza virus H1N1 indicated that the most potent extracts were the crude leaf extract (SI = 304.0) and the hexane fruits extract (SI = 358.0).

These most active extracts were then tested to determine the stage of the viral cycle at which the observed inhibitions was most pronounced. Interestingly, all tested extracts were shown to interfere with the virus infection step, when the virus was incubated directly with the extract before infecting the corresponding cell line. This pattern may suggest that the active extracts act at an early stage of infection, possibly by affecting viral particles or interfering with virus-cell interaction during adsorption or entry^51^. However, the present data do not allow confirmation of the exact molecular mechanism, and this possibility should therefore be interpreted with caution. Additional mechanistic studies would be required to determine whether these extracts interact directly with viral surface proteins, host cell receptors, or other factors involved in early infection. This more cautious interpretation is consistent with the plaque reduction findings and avoids overattributing the observed effect to a specific structural change in the virus. In silico docking studies were performed to determine which of the identified compounds in each extract are the most potent in binding to viral surface proteins, the HA and NA of influenza H1N1, and the spike proteins in SARS-CoV-2 and MERS-CoV. Interestingly, two chemical compounds, the lotoside II and hydroxygenistein methyl ether malonylhexoside, showed the most favorable docking profiles across all tested targets.

Lotoside II formed three hydrogen bonds with His38, Asn53, and Ser291 of the HA of H1N1. However, hydroxygenistein methyl ether malonylhexoside formed one hydrogen bond and one hydrogen-pi bond with His38. These residues are located in regions relevant to HA structure and function, site 38 in HA is located adjacent to the conserved stem epitope of the protein that directly controls virus replication rate^52^ and Asn53 is a conserved site that directly affects HA-mediated cell entry^53^, suggesting that these compounds may have the capacity to interact with biologically important sites. However, docking results alone cannot establish functional inhibition and should be interpreted only as supportive evidence for possible binding. Similarly, in the H1N1 NA model, lotoside II formed five hydrogen bonds with Asp151 (2), Trp178, Glu276, Glu277, and Arg371. However, hydroxygenistein methyl ether malonylhexoside formed two hydrogen bonds with Glu277 and Arg371. Asp151, Glu276, and Arg371 are the main conserved residues located in the catalytic active site of the NA protein, which are conserved in all influenza A NA subtypes^54,55^, while Trp178 and Glu277 are conserved structural residues in the active site of the NA protein^54^. Because several of these residues are located within or near the known active site of NA, these interactions may be relevant to protein binding. Nevertheless, it would be inappropriate to conclude from docking alone that binding to these amino acids directly inhibits protein function, viral replication, or viral release. Rather, these findings indicate that the identified compounds have predicted affinity for regions of functional interest, which supports their prioritization for future biochemical and target-based validation studies.

For the MERS-CoV spike protein, Lotoside II formed one hydrogen bond with Asp902, while hydroxygenistein methyl ether malonylhexoside bound to Asp902 and Glu944 with two hydrogen bonds. These two amino acids are located in the S2 region, which probably affects cell fusion and RNP release. The docking results of the SARS-CoV-2 spike protein showed that lotoside II formed three hydrogen bonds with Glu145, Glu402, and Arg518. Hydroxygenistein methyl ether malonylhexoside bound to Glu145, His345, and Ala348 with three hydrogen bonds, and formed two pi-pi interactions with His345 and Phe504. The 402 position is located in the active site of the cell receptor binding, which impairs proper binding to the hACE2 cell receptor. Position 145 in the N-terminal domain NTD directly affects viral replication rate^56^ and contributes to spike trimer assembly^57^. 345 and 348 residues are located in the heparin binding site in the receptor binding domain RBD, affecting the binding of the ACE2 receptor by controlling the opened or closed RBD position and exposure of RBD to the receptor^58^. However, as with the influenza targets, these docking results should be viewed as hypothesis-generating rather than confirmatory. They provide a computational basis for selecting candidate compounds for further study, but they do not demonstrate direct inhibition of receptor binding, fusion, or viral entry in the absence of orthogonal functional assays.

Taken together, the present findings show that *Ziziphus spina-christi* leaf and fruit extracts contain diverse bioactive constituents and exhibit measurable in vitro antiviral activity against H1N1, MERS-CoV, and SARS-CoV-2. Among the identified constituents, lotoside II and hydroxygenistein methyl ether malonylhexoside emerged as promising candidate compounds based on docking analysis. However, the contribution of these individual compounds to the observed extract-level antiviral activity remains to be experimentally confirmed. Therefore, while the current findings support the potential of *Ziziphus spina-christi* as a source of antiviral candidates, further studies are needed to isolate the active constituents, validate their molecular targets, and clarify their mechanisms of action in relevant biological systems.

## Conclusion

In conclusion, SARS-CoV-2 and influenza H1N1 continue to infect millions of people worldwide every year and impose substantial burdens on both health systems and economies. Plant-derived compounds and extracts have attracted considerable interest as potential antiviral agents because of their diverse bioactive properties, including possible direct antiviral effects and host-modulating activities. Ziziphus spina-christi extracts have previously been reported to possess several pharmacological properties, including anti-inflammatory and antimicrobial activities. In this study, the original leaf extract of Ziziphus spina-christi showed notable antiviral activity against H1N1 and SARS-CoV-2 in vitro. In addition, the residual leaf extract and the fruit hexane extract showed antiviral activity against MERS-CoV in both in vitro assays and in silico analyses. These findings suggest that Ziziphus spina-christi may represent a promising source of antiviral candidates against respiratory viruses. However, the present results do not support its use as a reference drug at this stage. Further studies are needed to isolate and characterize the active constituents, clarify their mechanisms of action, and evaluate their efficacy and safety in vivo and in preclinical models.

## Supplementary Information

Supplementary Material 1.

## Data Availability

The data will be available on request to the corresponding author.
