# Supplementary material for: Antiviral activity and chemical characterization of Egyptian Ziziphus spina-christi against human respiratory viruses
Source: Sci Rep. 2026 Apr 19;16:12749. doi: 10.1038/s41598-026-47325-9 (PMC13092631; doi:10.1038/s41598-026-47325-9)
Supplement: Supplementary file 1 — Supplementary Material 1. [file 41598_2026_47325_MOESM1_ESM.docx]

**SUPPORTING INFORMATION**

**Antiviral activity and chemical characterization of Egyptian *Ziziphus spina-christi* against human respiratory viruses**

Amany Elkhrsawy ^1,2^, Omnia Kutkat^1^, Yassmin Moatasim^1^, Mohamed S. Refaey^3,4^, Ahmed A. Al‐Karmalawy^5,6^, Eman Elabd^7^, Amr Keshta^2^, Kamal Shaltout^2^, Rabeh El-Shesheny^1,^ ^*^

Amany Elkhrsawy ^1,2^, Omnia Kutkat^1^, Yassmin Moatasim^1^, Mohamed S. Refaey^3,4^, Ahmed A. Al‐Karmalawy^5,6^, Eman Elabd^7^, Amr Keshta^2^, Kamal Shaltout^2^, Rabeh El-Shesheny^1,^ ^*^

^1^Centre of Scientific Excellence for Influenza Viruses, National Research Centre, Giza 12622, Egypt.

^2^Botany Department, Faculty of Science, Tanta University, 31527, Tanta, Egypt

^3^ Department of Pharmacognosy, Faculty of Pharmacy, University of Sadat City, Menoufia 32897, Egypt.

^4^Department of Pharmacognosy and Natural Products, Faculty of Pharmacy, Menoufia National University, km Cairo-Alexandria Agricultural Road, Menoufia, Egypt

^5^ Department of Pharmaceutical Chemistry, College of Pharmacy, the University of Mashreq, Baghdad 10023, Iraq.

^6^ Department of Pharmaceutical Chemistry, Faculty of Pharmacy, Horus University-Egypt, New Damietta 34518, Egypt. akarmalawy@horus.edu.eg

^7^ Department of Pharmaceutical and Drug Industries Research, National Research Center, Giza 12622, Egypt

^*^Corresponding authors at: Centre of Scientific Excellence for Influenza Viruses, National Research Centre, Giza, Egypt. E-mail address: [rabeh.elshesheny@human-linl.org](mailto:rabeh.elshesheny@human-linl.org)

**Content**

| **No.** | | **Contents** | **Page** | |
| --- | --- | --- | --- | --- |
| **Abbreviation list** | | |  | |
| **Table S1** | Metabolites tentatively identified from the ethyl acetate fraction of *Z. spina-christi* fruits using LC-ESI-MS/MS analysis in negative mode. | | | S3 |
| **Table S2** | Metabolites tentatively identified from the crude methanol extract of *Z. spina-christi* fruits using LC-ESI-MS/MS analysis in negative mode. | | | S6 |
| **Table S3** | Metabolites tentatively identified from the remaining aqueous fraction of *Z. spina-christi* fruits using LC-ESI-MS/MS analysis in negative mode. | | | S9 |
| **Table S4** | Metabolites tentatively identified from the crude methanol extract of *Z.*  *spina-christi* leaves using LC-ESI-MS/MS analysis in negative mode. | | | S11 |
| **Table S5** | Metabolites tentatively identified from the ethyl acetate fraction of *Z. spina-christi* leaves using LC-ESI-MS/MS analysis in negative mode | | | S14 |
| **Table S6** | Metabolites tentatively identified from the remaining aqueous fraction of *Z. spina-christi* fruits using LC-ESI-MS/MS analysis in negative mode | | | S9 |
| **Fig. S1** | Chemical structures of identified compounds in the ethyl acetate extract of *Z. spina christi* fruit. | | | S5 |
| **Fig. S2** | Chemical structures of identified compounds in crude methanol extract of *Z. spina christi* fruit. | | | S8 |
| **Fig. S3** | Chemical structures of identified compounds in remaining aqueous fraction of Z. spina christi fruit. | | | S10 |
| **Fig. S4** | Chemical structures of identified compounds in crude methanol extract of *Z. spina christi* leaves. | | | S13 |
| **Fig. S5** | Chemical structures of identified compounds in the ethyl acetate fraction of *Z. spina christi* leaves. | | | S15 |
| **Fig. S6** | Chemical structures of identified compounds in remaining aqueous fraction of *Z. spina christi* leaves. | | | S17 |
| **Fig. S7** | Superimposition of the redocked co-crystallized inhibitor (represented in green) over the native one (represented in red) for HA (PDB ID: 6WCR) and NA (PDB ID: 3CKZ) target receptors | | | S18 |
|  | References | | | S19 |

**Table S**1: Metabolites tentatively identified from the ethyl acetate fraction of Z. spina-christi fruits using LC-ESI-MS/MS analysis in negative mode.

| **R(min)** | **[M – H]^-^** | **Fragments ion (m/z)** | **Molecular formula** | **Tentative identification** | **References** |
| --- | --- | --- | --- | --- | --- |
| 1.02 | 179.00 | 135.27, 134.04 | **C_9_H_8_O_4_** | Caffeic acid | ^1^ |
| 6.48 | 301.04 | 179.04, 151.08 | C_15_H_10_O_7_ | Quercetin | ^1^ |
| 13.11 | 579.08 | 301.56, 300.36, 270.96, 179.28 | C_26_H_28_O_15_ | Quercetin 3-O-(2-O-rhamnosyl-arabinoside) | ^2^ |
| 9.04 | 463.03 | 301.08, 300.12, 150.96 | C_27_H_30_O_16_ | Quercetin-3-*O*-glucoside (isoquercetin) | ^1^ |
| 2.75 | 285.05 | 241.21, 201.12, 181.21, 149.04 | C_15_H_10_O_6_ | Luteolin | ^1^ |
| 22.42 | 303.15 | 151.23, 125.04 | C_15_H_12_O_7_ | Dihydroquercetin (taxifolin) | ^1^ |
| 25.82 | 547.16 | 503.16, 299.16, 284.16 | C_25_H_24_O_14_ | Hydroxygenistein methyl ether malonylhexosid | ^1^ |
| 17.23 | 177.01 | 149.04, 133.08, 105.69 | C_9_H_6_O_4_ | Dihydroxycoumarin I | ^1^ |
| 5.25 | 343.0988 | 181.08, 125.16 | C_18_H_18_O_7_ | 5,7-Dihydroxy- 8,3',5' trimethoxy-flavone | ^3^ |
| 26.56 | 397.22 | 325.44 | C_21_H_34_O_7_ | Stephanol | ^3^ |
| 13.11 | 579.08 | 301.56 | C_26_H_27_O_15_ | Quercetin-3-*O*-α-Larabinopyranosyl-(1→2)-α-L-rhamnopyranoside | ^4^ |
| 11.88 | 957.19 | 747.28, 911.28 | C_48_H_79_O_19_ | Lotoside II | ^5^ |
| 10.46 | 943.25 | 897.24 | C_48_H_79_O_18_ | Lotoside III | ^6^ |
| 11.69 | 485.18 | 423.24 | [C_30_H_46_O_5_](https://pubchem.ncbi.nlm.nih.gov/#query=C30H46O5) | Ceanothic acid | ^4^ |
| 12.06 | 839.22 | 675.24, 513.24, 348.24 | C_44_H_71_O_15_ | 15-Acetoxy-lotoside IV | ^6^ |
| 11.88 | 911.23 | 749.28, 603.19 | C_47_H_76_O_17_ | Zizyphus saponin I | ^7^ |
| 20.80 | 455.20 | 411.36 | C_30_H_47_O_3_ | Betulinic acid | ^7^ |

Fig. S**1**: Chemical structures of identified compounds in the ethyl acetate extract of *Z. spina christi* fruit.

Table S**2**: Metabolites tentatively identified from the crude methanol extract of *Z. spina-christi* fruits using LC-ESI-MS/MS analysis in negative mode.

| **R_t_**  **(min)** | **[M – H]^-^** | **Fragments ion (m/z)** | **Molecular formula** | **Tentative identification** | **Referance** |
| --- | --- | --- | --- | --- | --- |
| 1.10 | 179.01 | 135.61, 134.04 | **C_9_H_8_O_4_** | Caffeic acid | ^1^ |
| 1.11 | 341.01 | 179.16, 161.04, 135.12 | [C_15_H_18_O_9_](https://pubchem.ncbi.nlm.nih.gov/#query=C30H46O5) | Caffeic acid 4-*O*-glucoside | ^2^ |
| 6.40 | 301.12 | 179.04, 150.96 | C_15_H_10_O_7_ | Quercetin | ^1^ |
| 9.13 | 177.02 | 149.16, 133.08, 105.13 | C_9_H_6_O_4_ | Dihydroxycoumarin I | ^1^ |
| 11.94 | 957.18 | 911.28 | C_48_H_79_O_19_ | Lotoside II | ^5^ |
| 12.4 | 911.22 | 765.12, 749.49, 603.24 | C_47_H_76_O_17_ | Zizyphus saponin I | ^7^ |
| 12.57 | 485.18 | 423.24 | [C_30_H_46_O_5_](https://pubchem.ncbi.nlm.nih.gov/#query=C30H46O5) | Ceanothic acid | ^4^ |
| 12.57 | 485.18 | 423.24 | C_30_H_45_O_5_ | Epiceanothic acid | ^7^ |
| 15.28 | 397.10 | 325.21 | C_21_H_34_O_7_ | Stephanol | ^3^ |
| 16.94 | 353.12 | 191.16 | [C_16_H_18_O_9_](https://pubchem.ncbi.nlm.nih.gov/#query=C30H46O5) | *trans*-5-*O*-Caffeoylquinic acid | ^2^ |
| 20.75 | 455.20 | 411.36 | C_30_H_47_O_3_ | Betulinic acid | ^7^ |
| 20.8 | 453.20 | 407.16 | C_29_H_41_O_4_ | Ceanothenic acid | ^7^ |
| 22.97 | 353.13 | 191.04, 111.12 | [C_16_H_18_O_9_](https://pubchem.ncbi.nlm.nih.gov/#query=C30H46O5) | 3-*O*-Caffeoylquinic acid | ^2^ |
| 23.11 | 515.15 | 353.16 | [C_25_H_24_O_12_](https://pubchem.ncbi.nlm.nih.gov/#query=C30H46O5) | 4, 5-di-*O*-Caffeoylquinic acid | ^2^ |
| 25.80 | 547.17 | 299.28, 284.16 | C_25_H_24_O_14_ | Hydroxygenistein methyl ether malonylhexoside | ^1^ |

Fig. S**2**: Chemical structures of identified compounds in crude methanol extract of *Z. spina christi* fruit.

Table S**3**: Metabolites tentatively identified from the remaining aqueous fraction of *Z. spina-christi* fruits using LC-ESI-MS/MS analysis in negative mode.

| **R_t_**  **(min)** | **[M – H]^-^** | **Fragments ion (m/z)** | **Molecular formula** | **Tentative identification** | **References** |
| --- | --- | --- | --- | --- | --- |
| 1.12 | 341.01 | 178.92, 161.16, 134.88 | [C_15_H_18_O_9_](https://pubchem.ncbi.nlm.nih.gov/#query=C30H46O5) | Caffeic acid 4-*O*-glucoside | ^2^ |
| 1.14 | 179.01 | 135.12 | **C_9_H_8_O_4_** | Caffeic acid | ^1^ |
| 2.80 | 431.01 | 179.16 | C_22_H_24_O_09_ | Medicarpin 3-O-glucoside | ^3^ |
| 3.13 | 593.03 | 431.04, 269.16 | C_27_H_30_O_15_ | Apigenin 7, 4'-di-*O*-glucoside | ^2^ |
| 11.69 | 485.17 | 423.36 | C_30_H_45_O_5_ | Epiceanothic acid | ^7^ |
| 11.90 | 957.22 | 911.16 | C_48_H_79_O_19_ | Lotoside II | ^5^ |
| 11.99 | 911.21 | 748.92 | C_47_H_76_O_17_ | Zizyphus saponin I | ^7^ |
| 14.90 | 353.09 | 191.16 | [C_16_H_18_O_9_](https://pubchem.ncbi.nlm.nih.gov/#query=C30H46O5) | *trans*-5-*O*-Caffeoylquinic acid | ^2^ |
| 15.21 | 397.10 | 325.44 | C_21_H_34_O_7_ | Stephanol | ^3^ |
| 21.10 | 455.20 | 411.24 | C_30_H_47_O_3_ | Betulinic acid | ^7^ |
| 22.4 | 549.24 | 502.92, 221.28 | C_26_H_30_O_13_ | Jasminoside isomer | ^3^ |

Fig. S3: Chemical structures of identified compounds in remaining aqueous fraction of *Z. spina christi* fruit.

Table S**4**: Metabolites tentatively identified from the crude methanol extract of *Z. spina-christi* leaves using LC-ESI-MS/MS analysis in negative mode.

| **R_t_**  **(min)** | **[M – H]^-^** | **Fragments ion (m/z)** | **Molecular formula** | **Tentative identification** | **References** |
| --- | --- | --- | --- | --- | --- |
| 1.04 | 179.01 | 135.14, 134.04 | **C_9_H_8_O_4_** | Caffeic acid | ^1^ |
| 1.11 | 341.01 | 179.16, 161.04, 135.12 | [C_15_H_18_O_9_](https://pubchem.ncbi.nlm.nih.gov/#query=C30H46O5) | Caffeic acid 4-*O*-glucoside | ^2^ |
| 6.93 | 301.09 | 179.28, 151.08 | C_15_H_10_O_7_ | Quercetin | ^1^ |
| 11.50 | 485.21 | 423.36 | C_30_H_45_O_5_ | Epiceanothic acid | ^7^ |
| 7.45 | 177.01 | 149.04, 133.08, 105.63 | C_9_H_6_O_4_ | Dihydroxycoumarin I | ^1^ |
| 7.70 | 515.02 | 352.92 | [C_25_H_24_O_12_](https://pubchem.ncbi.nlm.nih.gov/#query=C30H46O5) | 4, 5-di-*O*-Caffeoylquinic acid | ^2^ |
| 11.88 | 957.18 | 911.28 | C_48_H_79_O_19_ | Lotoside II | ^5^ |
| 11.89 | 485.17 | 423.24 | [C_30_H_46_O_5_](https://pubchem.ncbi.nlm.nih.gov/#query=C30H46O5) | Ceanothic acid | ^4^ |
| 11.89 | 911.27 | 749.41, 603.36 | C_47_H_76_O_17_ | Zizyphus saponin I | ^7^ |
| 14.19 | 455.18 | 411.48 | C_30_H_47_O_3_ | Betulinic acid | ^7^ |
| 14.94 | 353.12 | 191.04, 179.16, 135.12 | [C_16_H_18_O_9_](https://pubchem.ncbi.nlm.nih.gov/#query=C30H46O5) | *cis-*5-*O*-Caffeoylquinic acid | ^2^ |
| 14.94 | 353.13 | 190.92, 108.96 | [C_16_H_18_O_9_](https://pubchem.ncbi.nlm.nih.gov/#query=C30H46O5) | *trans-*5-*O*-Caffeoylquinic acid | ^2^ |
| 16.70 | 453.16 | 407.64 | C_29_H_41_O_4_ | Ceanothenic acid | ^7^ |
| 25.81 | 547.17 | 299.41, 284.46 | C_25_H_24_O_14_ | Hydroxygenistein methyl ether malonylhexoside | ^1^ |

Fig. S4: Chemical structures of identified compounds in crude methanol extract of *Z. spina christi* leaves.

Table S**5**: Metabolites tentatively identified from the ethyl acetate fraction of *Z. spina-christi* leaves using LC-ESI-MS/MS analysis in negative mode.

| **R_t_**  **(min)** | **[M – H]^-^** | **Fragments ion (m/z)** | **Molecular formula** | **Tentative identification** | **Reference** |
| --- | --- | --- | --- | --- | --- |
| 1.40 | 179.00 | 135.85, 134.04 | **C_9_H_8_O_4_** | Caffeic acid | ^1^ |
| 6.64 | 301.11 | 179.04, 151.96 | C_15_H_10_O_7_ | Quercetin | ^1^ |
| 8.61 | 549.00 | 504.96, 179.04 | C_26_H_30_O_13_ | Jasminoside | ^3^ |
| 10.43 | 515.07 | 353.04 | [C_25_H_24_O_12_](https://pubchem.ncbi.nlm.nih.gov/#query=C30H46O5) | 4, 5-di-*O*-Caffeoylquinic acid | ^2^ |
| 11.24 | 485.18 | 423.36 | C_30_H_45_O_5_ | Epiceanothic acid | ^7^ |
| 11.98 | 957.17 | 911.16 | C_48_H_79_O_19_ | Lotoside II | ^5^ |
| 12.38 | 911.22 | 749.42, 603.24 | C_47_H_76_O_17_ | Zizyphus saponin I | ^7^ |
| 12.57 | 485.19 | 423.24 | [C_30_H_46_O_5_](https://pubchem.ncbi.nlm.nih.gov/#query=C30H46O5) | Ceanothic acid | ^4^ |
| 14.19 | 455.09 | 411.12 | C_30_H_47_O_3_ | Betulinic acid | ^7^ |
| 14.86 | 353.11 | 179.50, 135.96 | [C_16_H_18_O_9_](https://pubchem.ncbi.nlm.nih.gov/#query=C30H46O5) | *cis-*5-*O*-Caffeoylquinic acid | ^2^ |
| 20.46 | 453.21 | 407.16 | C_29_H_41_O_4_ | Ceanothenic acid | ^7^ |
| 25.81 | 547.18 | 299.16, 284.1646 | C_25_H_24_O_14_ | Hydroxygenistein methyl ether malonylhexoside | ^1^ |

Fig. S5: Chemical structures of identified compounds in the ethyl acetate fraction of *Z. spina christi* leaves.

Table S**6**: Metabolites tentatively identified from the remaining aqueous fraction of *Z. spina-christi* fruits using LC-ESI-MS/MS analysis in negative mode.

| **R_t_^a^**  **(min)** | **[M – H]^-^** | **Fragments ion (m/z)** | **Molecular formula** | **Tentative identification** | **Reference:** |
| --- | --- | --- | --- | --- | --- |
| 1.05 | 179.03 | 135.12, 134.04 | **C_9_H_8_O_4_** | Caffeic acid | ^1^ |
| 6.26 | 301.12 | 179.16, 151.08 | C_15_H_10_O_7_ | Quercetin | ^1^ |
| 11.65 | 485.18 | 423.36 | C_30_H_45_O_5_ | Epiceanothic acid | ^7^ |
| 11.97 | 911.22 | 749.28, 603.12 | C_47_H_76_O_17_ | Zizyphus saponin I | ^7^ |
| 17.91 | 453.15 | 407.28 | C_29_H_41_O_4_ | Ceanothenic acid | ^7^ |
| 26.60 | 547.18 | 299.28, 284.28 | C_25_H_24_O_14_ | Hydroxygenistein methyl ether malonylhexoside | ^1^ |
| 16.96 | 353.12 | 191.16 | [C_16_H_18_O_9_](https://pubchem.ncbi.nlm.nih.gov/#query=C30H46O5) | *trans*-5-*O*-Caffeoylquinic acid | ^2^ |
| 9.55 | 957.23 | 911.16 | C_48_H_79_O_19_ | Lotoside II | ^5^ |
| 21.08 | 455.20 | 411.36 | C_30_H_47_O_3_ | Betulinic acid | ^7^ |

Fig. S**6**: Chemical structures of identified compounds in remaining aqueous fraction of *Z. spina christi* leaves.

| **HA (PDB ID: 6WCR)** | **NA (PDB ID: 3CKZ)** |
| --- | --- |
| 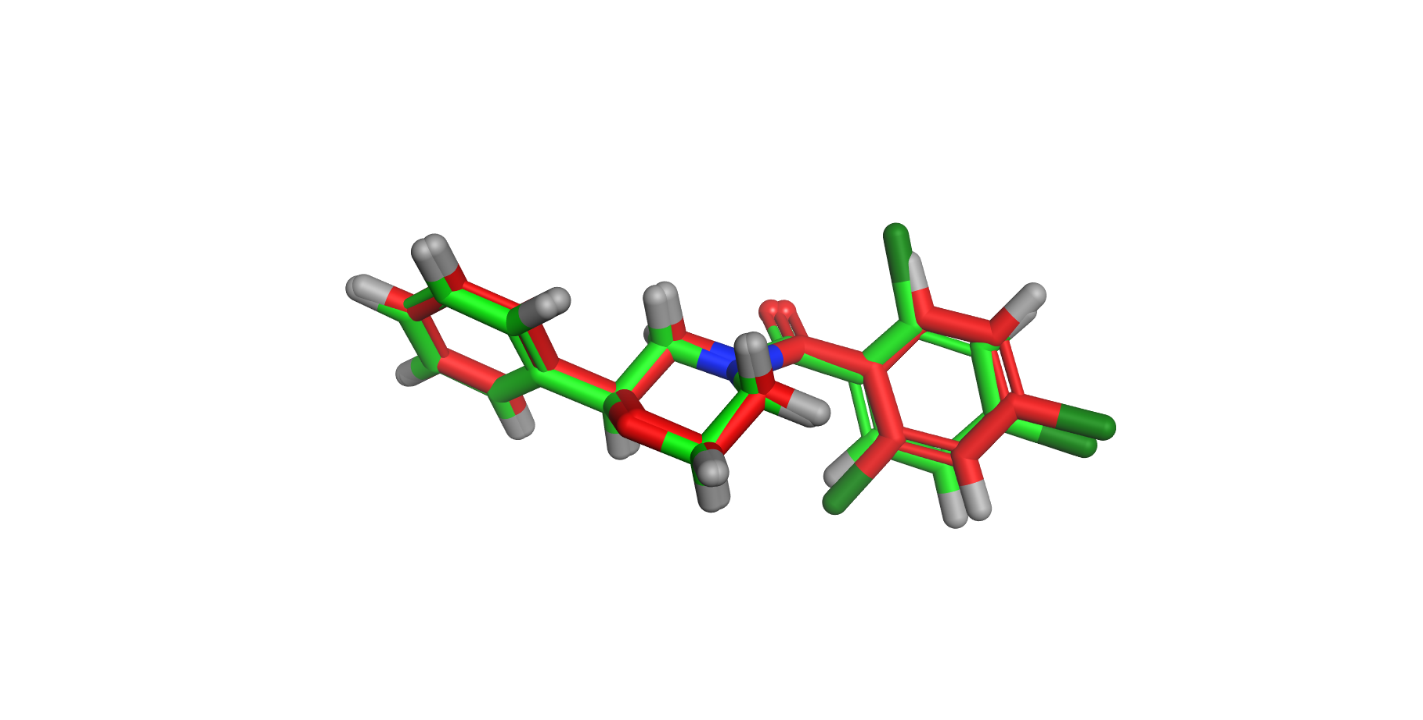 | 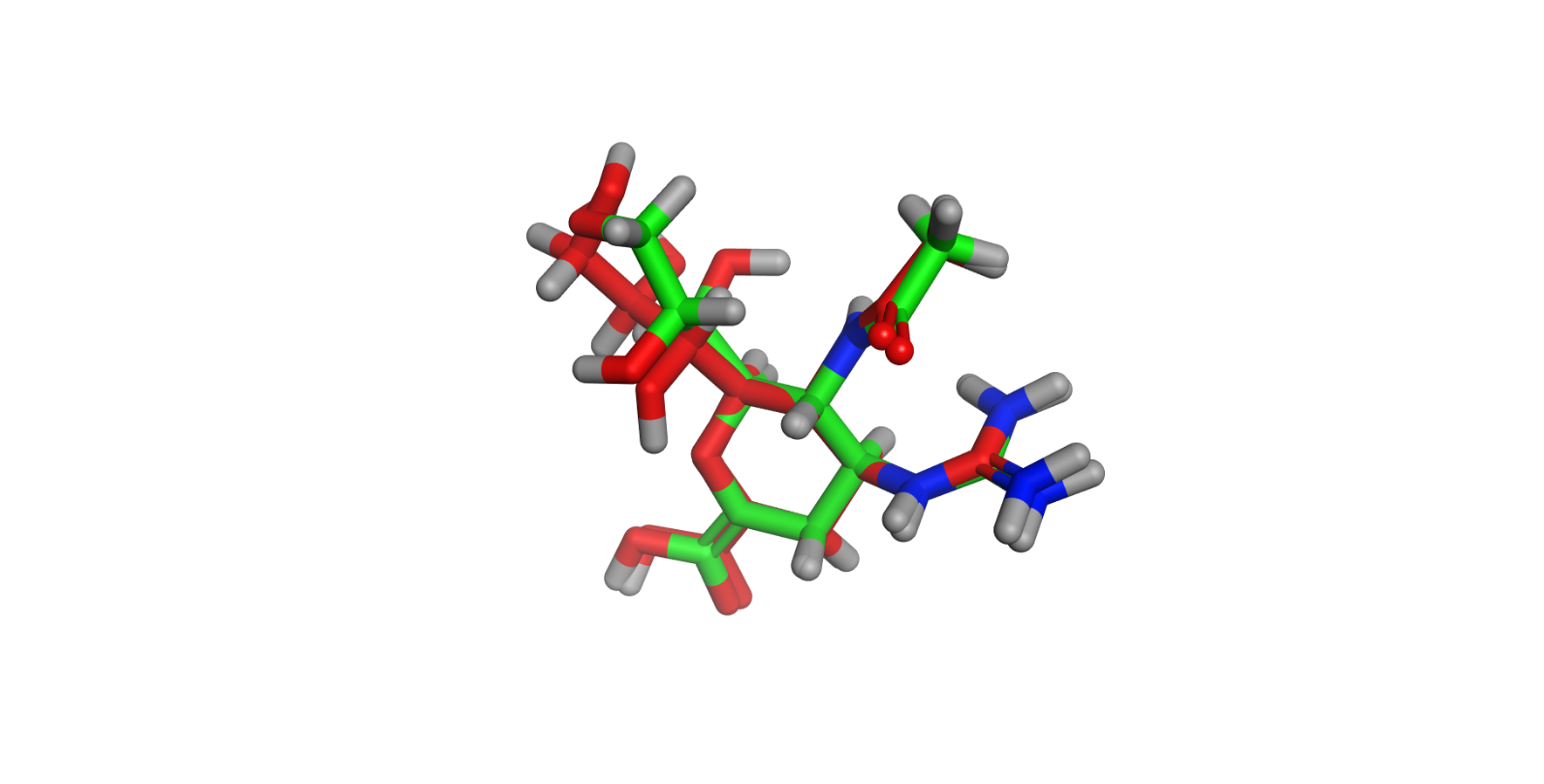 |

Fig. S**7**: Superimposition of the redocked co-crystallized inhibitor (represented in green) over the native one (represented in red) for HA (PDB ID: 6WCR) and NA (PDB ID: 3CKZ) target receptors

**Reference:**

1 Ammar, S., del Mar Contreras, M., Belguith-Hadrich, O., Bouaziz, M. & Segura-Carretero, A. New insights into the qualitative phenolic profile of Ficus carica L. fruits and leaves from Tunisia using ultra-high-performance liquid chromatography coupled to quadrupole-time-of-flight mass spectrometry and their antioxidant activity. *RSC Advances* **5**, 20035-20050 (2015).

2 Karar, M. E. *et al.* Phenolic profile and in vitro assessment of cytotoxicity and antibacterial activity of Ziziphus spina-christi leaf extracts. *Med chem* **6**, 143-156 (2016).

3 Berkani, F. *et al.* New bioactive constituents characterized by LC–MS/MS in optimized microwave extract of jujube seeds (Zizyphus lotus L.). *Journal of Food Measurement and Characterization* **15**, 3216-3233 (2021).

4 Guo, S. *et al.* Simultaneous qualitative and quantitative analysis of triterpenic acids, saponins and flavonoids in the leaves of two Ziziphus species by HPLC–PDA–MS/ELSD. *Journal of pharmaceutical and biomedical analysis* **56**, 264-270 (2011).

5 Wang, S. *et al.* Identification of chemical constituents in the extract and rat serum from Ziziphus jujuba mill by HPLC-PDA-ESI-MSn. *Iranian journal of pharmaceutical research: IJPR* **13**, 1055 (2014).

6 Bozicevic, A., De Mieri, M., Di Benedetto, A., Gafner, F. & Hamburger, M. Dammarane-type saponins from leaves of Ziziphus spina-christi. *Phytochemistry* **138**, 134-144 (2017).

7 Masullo, M., Cerulli, A., Montoro, P., Pizza, C. & Piacente, S. In depth LC-ESIMSn-guided phytochemical analysis of Ziziphus jujuba Mill. leaves. *Phytochemistry* **159**, 148-158 (2019).

| **HA (PDB ID: 6WCR)** | **NA (PDB ID: 3CKZ)** |
| --- | --- |
| 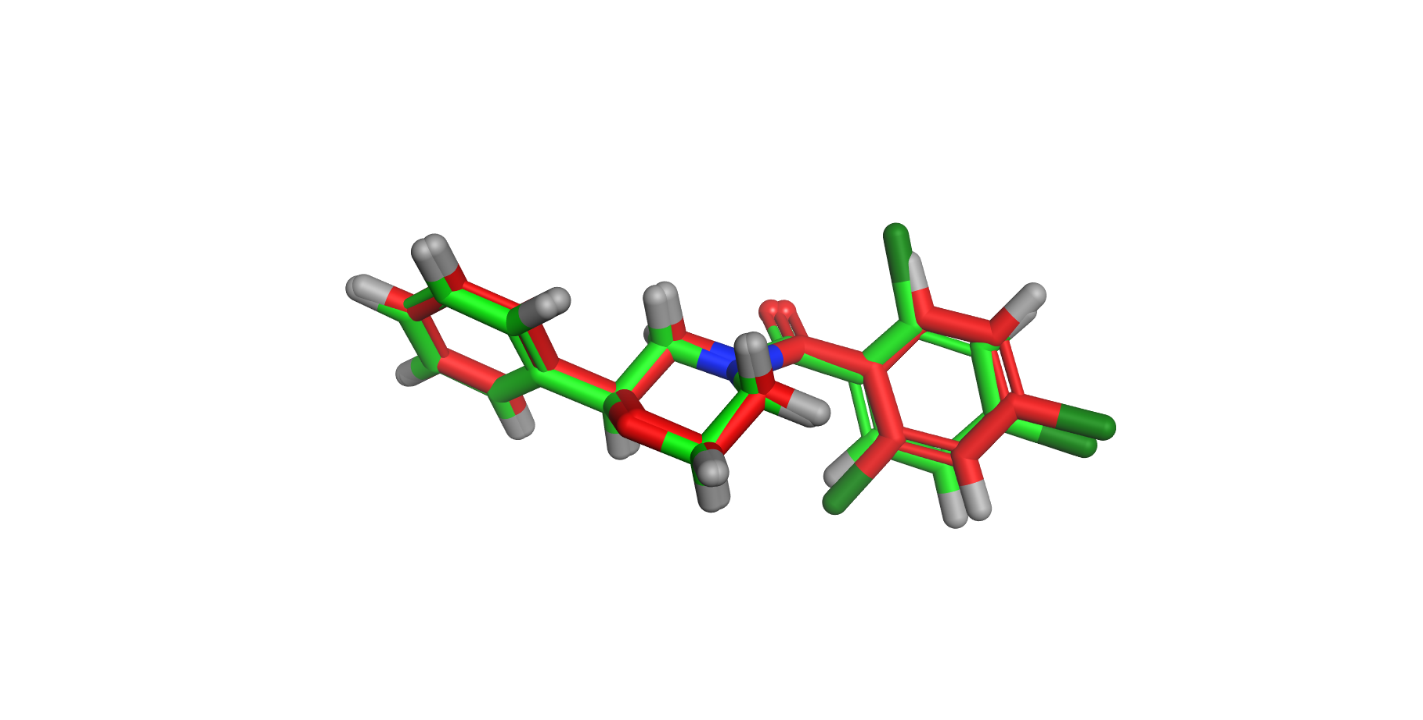 | 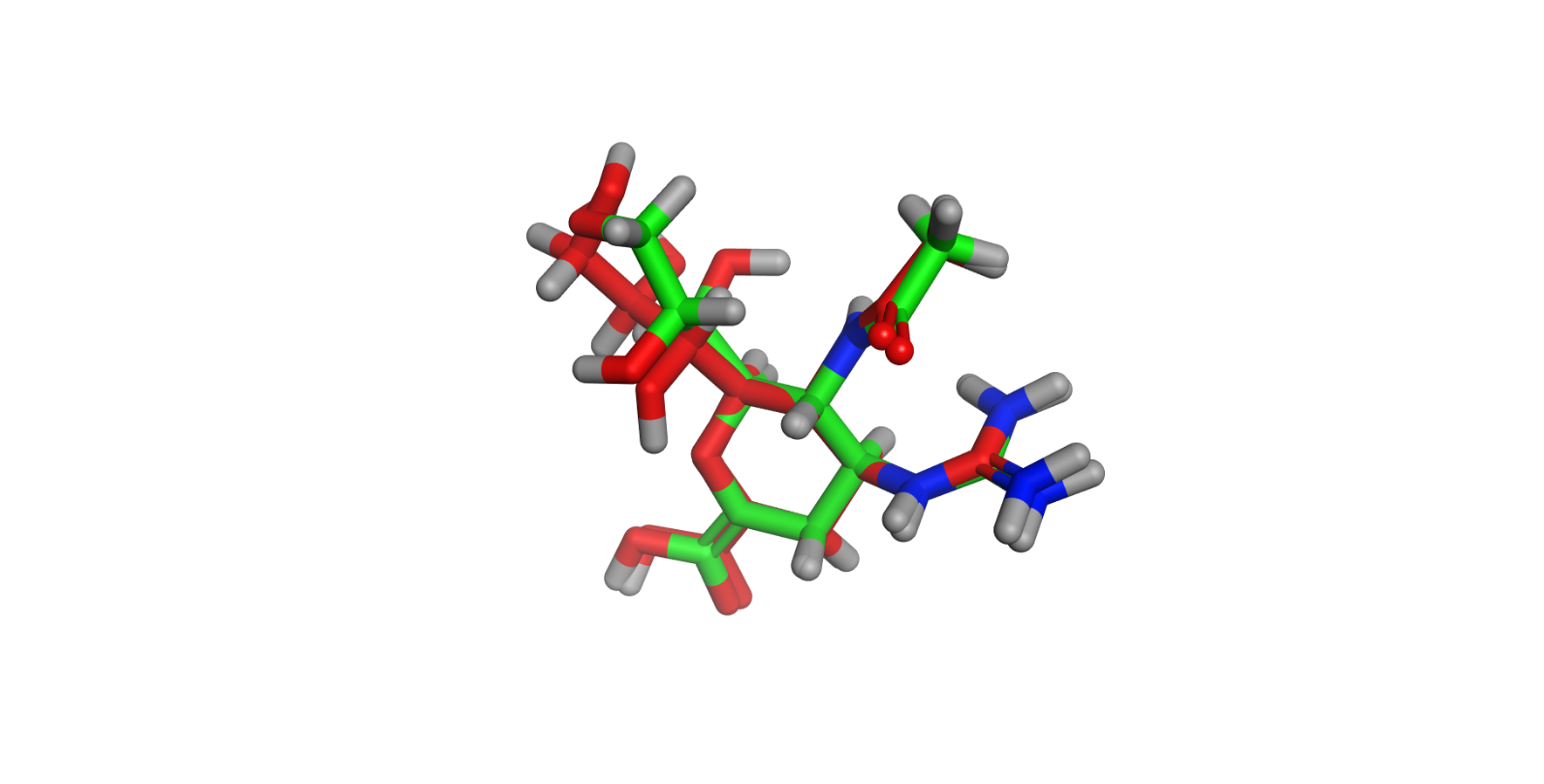 |

Figure S7: Superimposition of the redocked co-crystallized inhibitor (represented in green) over the native one (represented in red) for HA (PDB ID: 6WCR) and NA (PDB ID: 3CKZ) target receptors.
